# The chromosome-scale genome reveals the evolution and diversification after the recent tetraploidization event in tea plant

**DOI:** 10.1038/s41438-020-0288-2

**Published:** 2020-05-01

**Authors:** Jie-Dan Chen, Chao Zheng, Jian-Qiang Ma, Chen-Kai Jiang, Sezai Ercisli, Ming-Zhe Yao, Liang Chen

**Affiliations:** 1 Key Laboratory of Tea Biology and Resources Utilization, Ministry of Agriculture and Rural Affairs, Tea Research Institute of the Chinese Academy of Agricultural Science, Hangzhou, 310008 China; 2 Department of Horticulture, Faculty of Agriculture, Ataturk University, Erzurum, Turkey

**Keywords:** Genome evolution, Comparative genomics

## Abstract

Tea is one of the most popular nonalcoholic beverages due to its characteristic secondary metabolites with numerous health benefits. Although two draft genomes of tea plant (*Camellia sinensis*) have been published recently, the lack of chromosome-scale assembly hampers the understanding of the fundamental genomic architecture of tea plant and potential improvement. Here, we performed a genome-wide chromosome conformation capture technique (Hi-C) to obtain a chromosome-scale assembly based on the draft genome of *C. sinensis* var. *sinensis* and successfully ordered 2984.7 Mb (94.7%) scaffolds into 15 chromosomes. The scaffold N50 of the improved genome was 218.1 Mb, ~157-fold higher than that of the draft genome. Collinearity comparison of genome sequences and two genetic maps validated the high contiguity and accuracy of the chromosome-scale assembly. We clarified that only one *Camellia* recent tetraploidization event (CRT, 58.9–61.7 million years ago (Mya)) occurred after the core-eudicot common hexaploidization event (146.6–152.7 Mya). Meanwhile, 9243 genes (28.6%) occurred in tandem duplication, and most of these expanded after the CRT event. These gene duplicates increased functionally divergent genes that play important roles in tea-specific biosynthesis or stress response. Sixty-four catechin- and caffeine-related quantitative trait loci (QTLs) were anchored to chromosome assembly. Of these, two catechin-related QTL hotspots were derived from the CRT event, which illustrated that polyploidy has played a dramatic role in the diversification of tea germplasms. The availability of a chromosome-scale genome of tea plant holds great promise for the understanding of genome evolution and the discovery of novel genes contributing to agronomically beneficial traits in future breeding programs.

## Introduction

Tea, one of the most popular nonalcoholic beverages in the world, provides characteristic secondary metabolites, such as catechins, theanine, and caffeine that have numerous health benefits for humans^1–3^. The tea plant (*Camellia sinensis* (L.) O. Kuntze) originated in southwest China and has expanded worldwide to >50 countries^4–6^. By 2018, the worldwide cultivated area and production of tea have increased to 4.9 million hectares and 5.9 million tons, respectively^7^. Tea has created economic benefits and vast employment opportunities, especially in some Asian and African countries, such as China, India, Sri Lanka, and Kenya. In addition, due to distinctive sets of secondary metabolites (such as various catechins, caffeine, and theanine), tea has been widely applied in expounding the molecular mechanisms regulating catechins and theanine biosynthesis^8–12^. However, the lack of a high-quality genome sequence has become the main hindrance to gaining insights into secondary metabolite biosynthesis and fully understanding the evolution of tea plant. High levels of heterozygosity and repetitiveness pose a challenge to genome assembly in tea plant. Recently, two draft genomes of tea plant have been published by Illumina next-generation sequencing technology, but these genomes remain highly fragmented (scaffold N50: 449.5 kb in *C. sinensis* var. *assamica* genome and 1.4 Mb in *C. sinensis* var. *sinensis* (CSS) genome) and need improved completion^13–15^.

Chromosome-scale genome assembly is essential for genome-wide association study (GWAS) and the identification of quantitative trait loci (QTLs) governing important agronomic traits to facilitate gene cloning^16^. A high-quality reference genome also accelerates genome evolution involving ancient whole-genome duplications (WGDs), segmental duplications, tandem duplications, structural evolution, etc. The traditional BAC-by-BAC approach, integrating genetic and physical maps, has been used to produce high-quality assemblies^17,18^. However, this approach remains prohibitively expensive and laborious, which are bottlenecks for its widespread application to genome assembly. A genetic linkage map is another approach that assigns contigs or scaffolds to chromosomes^19–21^. A high-density SLAF-seq (SNP and SSR) genetic map of tea plant has been used to anchor scaffolds to chromosomes in the draft genome of CSS^13,22^. However, it is often difficult to generate a high-quality genome sequence, especially in centromeric regions, depending on the density of molecular markers, mapping population, and recombination events. Recently, Hi-C (high-throughput/resolution chromosome conformation capture), an effective and efficient approach, has been developed to guide genome assembly^23–25^. Due to the highly folded structures of chromosomes in a cell, sequences that are spatially proximal in three-dimensional (3D) space can be far apart along the linear chromosome. Hi-C detects spatially proximal DNA interactions by a proximity ligation method combined with Illumina sequencing. The frequency of contact between paired-end reads of loci can infer the distance of two DNA fragments, and further anchor and orient scaffolds. Hi-C has become a powerful approach for generating high-quality genome assemblies and has been widely performed in the genome assembly of animals and plants, such as humans^24^, *Arabidopsis*^26^, and black raspberry^27^.

WGD, also known as polyploidy, has been widely detected in plants and is considered an important evolutionary force in plants^28,29^. The WGD events are followed by chromosomal rearrangement, gene loss of most duplicates, and duplicate gene expression bias, causing a dramatic increase in species richness in angiosperms^30,31^. After the divergence of eudicots and monocots, a hexaploidization event (γ event) shared by all core eudicots occurred 140 million years ago (Mya)^32^. Because the *Vitis vinifera* genome had no additional WGD event except the hexaploidization event and preserved the ancestral eudicot chromosome structure, it has been widely used as a reference genome for the study of evolution^33–35^. *Actinidia chinensis*, the closest genus to *Camellia* in phylogenetic trees, showed two additional tetraploidization events after the hexaploidization event, namely, the *Actinidia* recent tetraploidization at ~18–20 Mya and the *Actinidia* ancient tetraploidization at ~50–57 Mya^35,36^. With the completion of the draft genome sequences of *C. sinensis*, two opinions regarding WGDs in the *C. sinensis* genome appeared: that two additional rounds of WGD events occurred in *C. sinensis* after the hexaploidization event^13^ and that only one additional WGD event occurred in *C. sinensis* independent with tetraploidization in *A. chinensis*^14,36^. Tandem duplicates that arise by unequal crossing over are closely adjacent identical genes in the same chromosome and are prevalent in eukaryotes^37^. Tandem duplicates usually tend to share common functions owing to co-regulated elements^38^. However, it has been revealed that some tandem duplicates have generated novel functions by acquiring novel transcription patterns^39^. Moreover, tandem duplicates play an important role in adaptive evolution to rapidly changing environments^40^.

In this study, Hi-C analysis of CSS ‘Shuchazao’ was performed to improve the draft genome sequence of CSS, generating a chromosome-scale assembly for tea plant. Based on the chromosome-scale genome, WGD and tandem duplication were identified to investigate recursive polyploidizations and diversification of duplicate genes. Furthermore, the published QTLs related to the catechins and caffeine content in tea were integrated into the chromosome-scale genome to facilitate the identification of the effective genes. These results improved our understanding of the evolution and diversification of duplicated genes in tea plant, laying a substantial foundation for the discovery of novel genes contributing to agronomically beneficial traits in future breeding programs.

## Results

### Chromosome-scale assembly of CSS

**Fig. 1:**
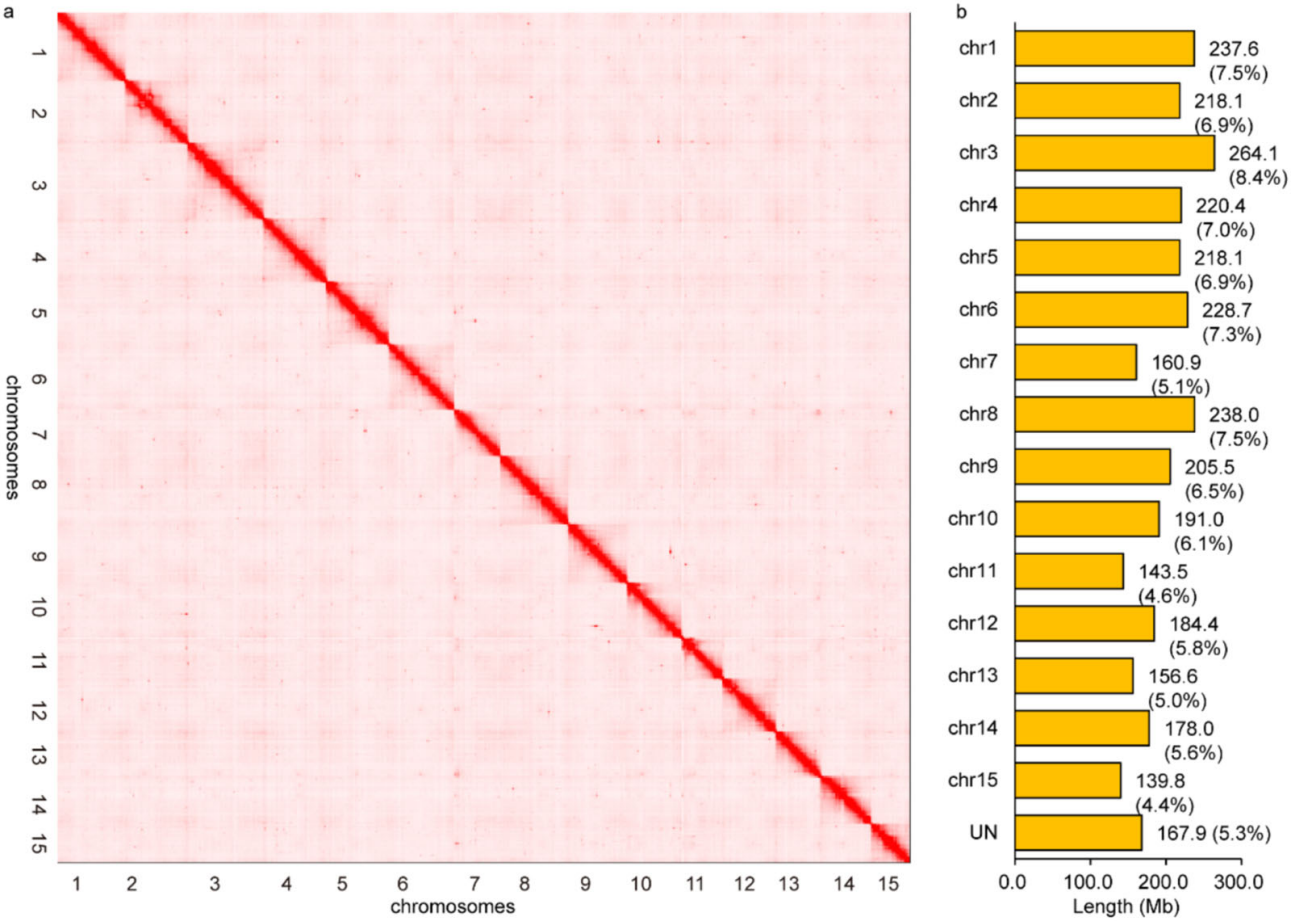
Hi-C heatmap based on the chromosome-scale assembly of the CSS V1.2 genome. **a** The heatmap represents the contact matrices generated by aligning the Hi-C data to the chromosome-scale assembly of the CSS V1.2 genome. **b** The length statistics of each chromosome of the CSS V1.2 genome resulting from the Juicer and 3D-DNA pipelines

To obtain a chromosome-scale assembly of tea plant, a total of 1,126,108,661 Hi-C read pairs of CSS ‘Shuchazao’ (337.8 Gb, ~113-fold genome coverage) were generated and mapped to the draft genome of CSS^13^. After the removal of erroneous mappings and PCR duplicates, the remaining 507,043,204 read pairs (45.0% of total reads) were used to construct Hi-C linking information and a Hi-C scaffolding pipeline (misjoin correction, ordering, and orientation). Finally, the chromosome-scale assembly of the CSS V1.2 genome spanning 3.2 Gb of genome sequence was generated. In this study, the 2984.7 Mb genome sequence was clustered into 15 superscaffolds, accounting for 94.7% of the total genome size. The scaffold N50 of the CSS V1.2 genome was 218.1 Mb, ~157-fold higher than that of the draft genome (1.4 Mb, Supplementary Table 1). The Hi-C heatmap showed that 15 superscaffolds in the CSS V1.2 genome could be distinguished and perfectly represented 15 chromosomes (Fig. 1a). The chromosome names from chr1 to chr15 were defined by the previous genetic map from LG01 to LG15, respectively^22^. Of the 15 chromosomes, the longest and shortest assembled pseudomolecules were chr3 and chr15 at 264,061,170 and 139,796,614 bp, respectively, and the average length was 198,979,785 bp (Fig. 1b). To facilitate identification of functional genes, 32,311 protein-coding genes (95.2% of annotated genes in the draft genome) were anchored to the chromosomes of the CSS V1.2 genome. The number of genes on each chromosome varied significantly from 1467 to 3013 (mean, 2154) and showed that the gene number was correlated to chromosome size (*r*^2^ = 0.91, Pearson correlation coefficient). The size of transposable elements (TEs) had a higher coefficient of association (*r*^2^ = 0.99) than the number of genes. However, the proportion of TEs in each chromosome remained relatively constant, from 55.5% to 59.5% (mean, 57.4%, Supplementary Table 2).

**Fig. 2:**
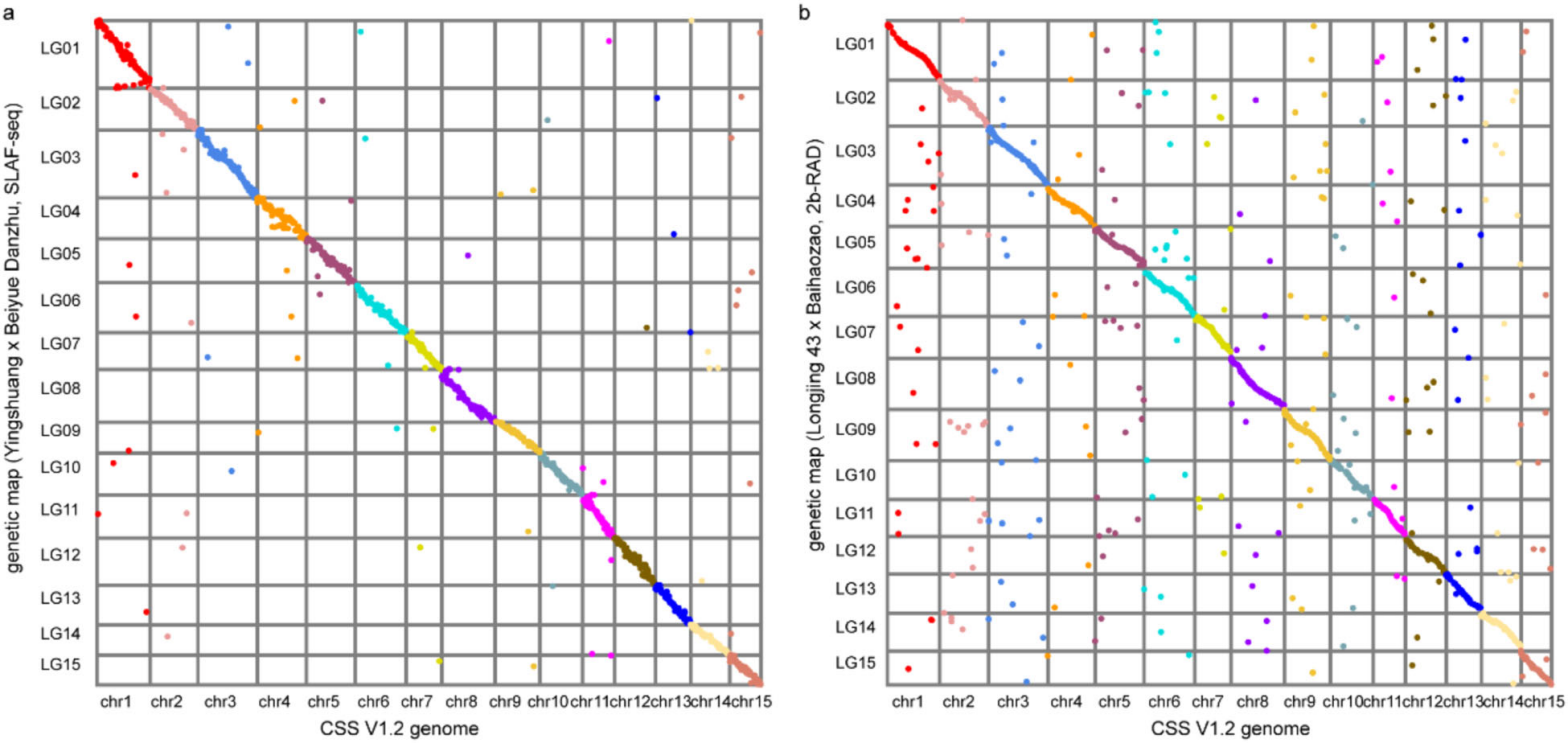
Comparison of the chromosome-scale assembly of the CSS V1.2 genome and genetic maps. **a** The *y*-axis represents the genetic positions of the SLAF-seq genetic map from the F1 population of CSS ‘Yingshuang’ and *C. sinensis* var. *pubilimba* ‘Beiyue Danzhu’. **b** The *y*-axis represents the genetic positions of the 2b-RAD genetic map from the F1 population of CSS ‘Longjing 43’ and CSS ‘Baihaozao’. The *x*-axis represents the physical positions of the chromosome-scale assembly of the CSS V1.2 genome

To assess the improved genome, 6042 and 2380 available markers from the SLAF-seq^22^ and 2b-RAD^41^ genetic maps were assigned to the CSS V1.2 genome. After filtering multimapped sequences, 2035 and 1828 unambiguous alignments in the SLAF-seq and 2b-RAD genetic maps were retained, and 96.9% and 83.1% of these were correctly identified in the CSS V1.2 genome, respectively, in the sense that these markers appeared on the same chromosome in the genetic map and the CSS V1.2 genome (Supplementary Table 3). Collinearity comparison between the physical map and the genetic map validated the high contiguity and accuracy of the CSS V1.2 genome (Fig. 2).

### Whole-genome duplication

**Fig. 3:**
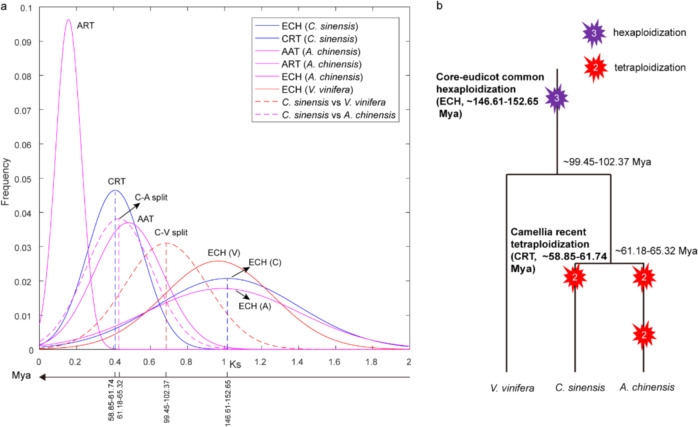
Dating of two polyploidization events of the *C. sinensis* genome. **a** *K*s distribution between collinear genes within the *C. sinensis* genome or among genomes. CRT *Camellia* recent tetraploidization, ECH core-eudicot common hexaploidization, ART *Actinidia* recent tetraploidization, AAT *Actinidia* ancient tetraploidization. **b** Phylogenetic tree of *C. sinensis*, *V. vinifera*, and *A. chinensis*

Two opinions, namely, that one or two additional WGD events in *C. sinensis* occurred after the hexaploidization event (γ event) shared by the eudicot genome, have been reported^13,14^. To clarify the question of how many rounds of WGD events occurred in *C. sinensis*, the sequence divergence of homologous genes and gene dotplots were used to infer ancient polyploidization events. First, self-comparison of the *C. sinensis* genome was carried out, and 254 homologous blocks containing 4748 gene pairs were identified in the intragenomic gene collinearity of *C. sinensis*. The synonymous nucleotide substitutions (*K*s) of these homologous gene pairs were calculated. These homologous blocks resulted from multiple polyploidization events, so we classified them by the median *K*s of each homologous block based on the phenomenon that *K*s of homologous genes produced by one polyploidization event were usually similar. The histogram plot with the median *K*s of homologous blocks showed two clearly distinguishable peaks (Supplementary Fig. 1), suggesting that two ancient polyploidization events exist in *C. sinensis*. The homologous blocks with relatively high *K*s values might have resulted from the core-eudicot common hexaploidization event (ECH), while other blocks (*K*s ≤ 0.7) might have been generated by the *Camellia* recent WGD event. The homologous gene dotplot within the *C. sinensis* genome integrated with the *K*s information further clarified that one *Camellia* recent tetraploidization event (CRT) occurred after the ECH event (Supplementary Fig. 2). These blocks were classified into 177 ECH-related and 77 CRT-related homologous blocks, containing 2152 (45.3%, 12.2 gene pairs per block) and 2596 (54.7%, 33.7) gene pairs, respectively, revealing that CRT has a more highly conserved synteny than ECH in *C. sinensis* (Supplementary Table 4). To estimate the occurrence times of two polyploidization events, curve fitting of the *K*s distribution of homologous genes resulting from the ECH and CRT events was performed. The peaks of the two polyploidization events were at 0.4 and 1.0, suggesting that the CRT and ECH events occurred at 58.9–61.7 and 146.6–152.7 Mya based on a neutral substitution rate of 3.39 × 10^−936^, respectively (Fig. 3a). Although the CRT event occurred near the divergence time of *C. sinensis* and *A. chinensis* from their common ancestor (61.2–65.3 Mya), a previous report indicates that the tetraploidization event was a lineage-specific WGD^14,36^ (Fig. 3b).

**Fig. 4:**
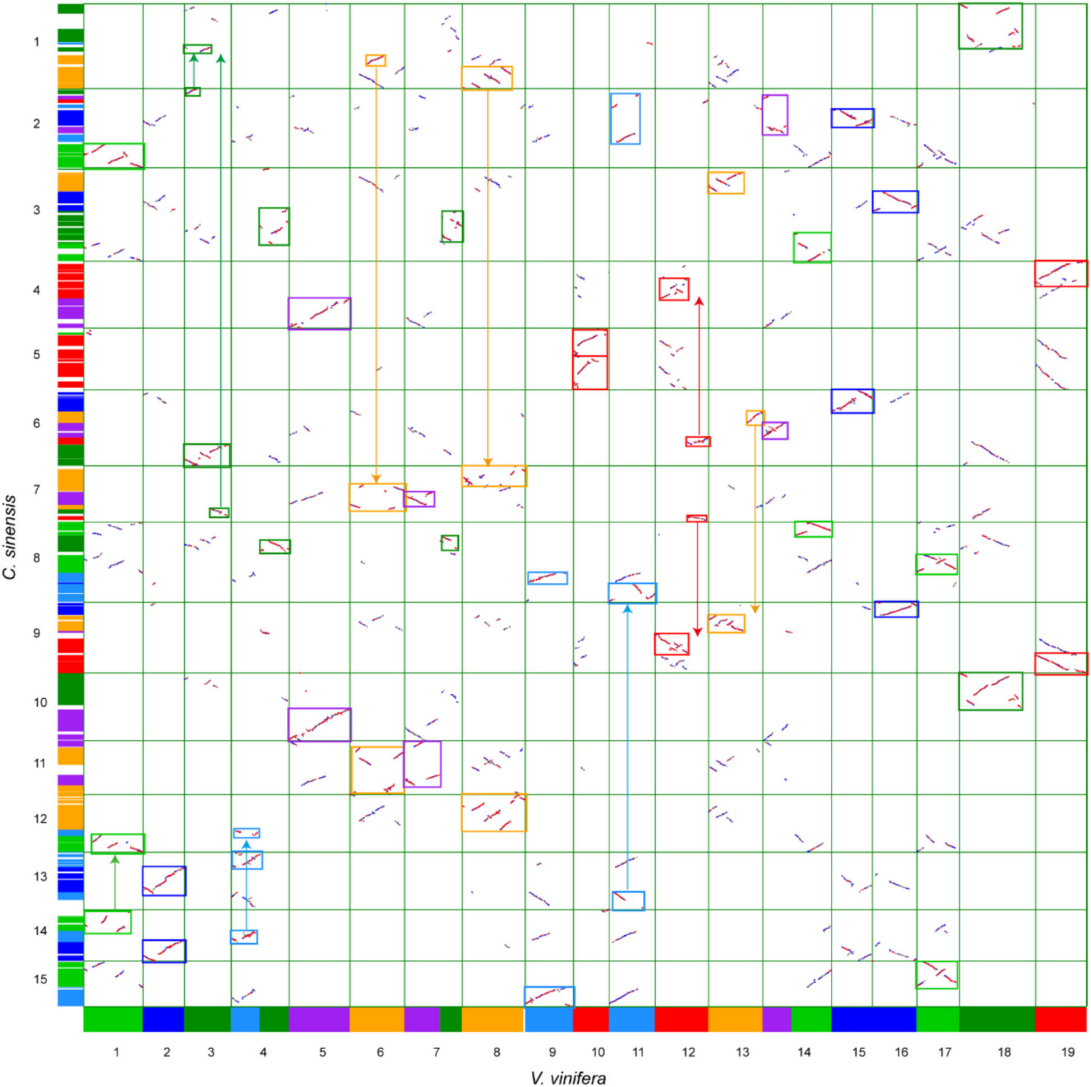
Homologous dotplot between *C. sinensis* and *V. vinifera* genomes. The red, blue, and gray dots represent the best, secondary, and other matched homologous gene pairs resulting from the output of Blast software. The 19 chromosomes of the *V. vinifera* genome are colored by the seven eudicot ancestral chromosomes. The orthologous regions were identified and marked by squares

To further verify two polyploidization events (ECH and CRT) in *C. sinensis*, the CSS V1.2 genome was compared with *V. vinifera*, known to be the closest relative to the eudicot ancestor structured. Based on the all-vs-all blastp analysis, 714 homologous blocks containing 14,831 gene pairs between *C. sinensis* and *V. vinifera* were identified (Supplementary Table 5). Using *V. vinifera* as the reference genome, for almost every *V. vinifera* chromosome, six collinear regions can be identified in the *C. sinensis* genome (Fig. 4). Among these, the best and second best-matched *V. vinifera* regions resulted from the CRT event and ECH event, respectively. Thus, this 1-to-2 relationship between *V. vinifera* and *C. sinensis* genomic regions inferred from the best-matching *V. vinifera* regions was a clear indication of a tetraploidization event in the *C. sinensis* genome after the split with *V. vinifera*. In addition, the following *C. sinensis* chromosome to *V. vinifera* chromosome correspondences were established (c for *C. sinensis* and v for *V. vinifera* as chromosome nomenclature): c1/v18-v8-v3-v6, c2/v1-v11-v15-v14-v3, c3/v13-v16-v14-v4-v7, c4/v5-v19-v12, c5/v10, c6/v3-v15-v14-v13-v12, c7/v8-v6-v7-v3-v12, c8/v11-v9-v17-v14-v7-v4, c9/v12-v19-v13-v16, c10/v5-v18, c11/v6-v7, c12/v8-v4-v1, c13/v2-v4-v11, c14/v1-v2-v4, c15/v9-v17. Among these, c5 was fused with its chromosomal segment duplications corresponding to v10. However, the fission and fusion of at least five chromosomes resulted in the longest chromosome c3.

Polyploidization events increased the complexity of the *C. sinensis* genome, including genome size, gene content, and gene expression. For example, *C. sinensis* chr5 mainly contained two intrachromosomal segmental duplications, suggesting that it fused with its homolog after the recent tetraploidization event, corresponding to chr10 in *V. vinifera*. Although 250 paralogous gene pairs exhibited substantially conserved synteny between chr5a (chr5:21.3–86.1 Mb) and chr5b (chr5:89.9–218.1 Mb), these genes accounted for only 25.4% and 35.6% of all genes in chr5a and chr5b, respectively, and most genes exhibited divergence between the two duplicates. Moreover, the segmental length of chr5b was twice that of chr5a, resulting from variation in the number of TEs during genome evolution and diversification (78.3 Mb TEs in chr5b and 36.4 Mb in chr5a, Supplementary Fig. 3a). Among these retained genes both in chr5a and chr5b, expression divergence between duplicate genes was widespread during evolution, and 50.5–57.8% of duplicate genes had differential expression in one of the eight tissues (Supplementary Fig. 3b). The duplicate genes of asymmetrical evolution in sequence, structure, and expression may be important for the evolution of the *C. sinensis* genome.

### Tandem gene duplication

**Fig. 5:**
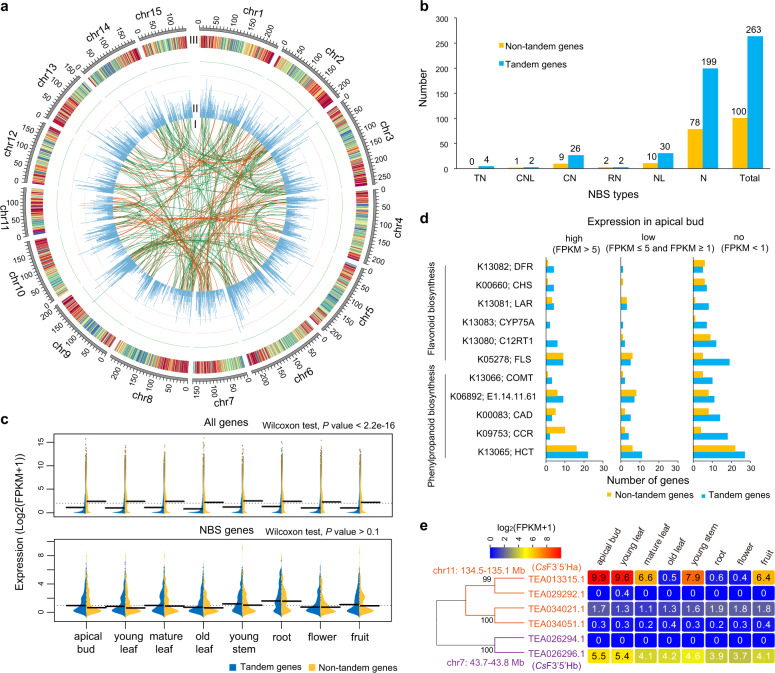
Genome-wide analysis of tandem gene duplication in the *C. sinensis genome*. **a** *C. sinensis* genome topography and synteny. I represents the duplicated gene blocks within *C. sinensis* that were produced by ECH (green) and CRT (orange) events. II represents the frequency distribution of tandem genes in 1-Mb intervals across the 15 chromosomes, MAX = 22. III represents gene density ranging from 0 to 37 genes per Mb. **b** Identification of tandemly duplicated NBS-encoding genes. **c** Comparison of gene expression in eight tissues between tandem and nontandem NBS-encoding genes. **d** Comparison of the highly expressed apical genes between tandem and nontandem genes among the enriched genes involved in phenylpropanoid biosynthesis and flavonoid biosynthesis. **e** Phylogenetic tree and gene expression in eight tissues of six F3ʹ5ʹH genes belonging to two clusters

Tandem duplication was also detected by BLASTP (*e*-value < 1e−20), with a maximum of five intervening genes. A total of 3262 tandem expanded regions were detected in the *C. sinensis* genome and were distributed unevenly on 15 chromosomes (Fig. 5a). The tandem expanded regions contained 9243 genes (28.6% of all genes on the chromosomes), which was higher than the numbers in *A. chinensis* (3111, 10.1%) and *V. vinifera* (5088, 21.5%, Supplementary Table 6). The most tandem duplicates in the *C. sinensis* genome were the result of a steady and unusually high rate of tandem duplicate gain after the recent tetraploidization event (Supplementary Fig. 4). The tandem genes had significantly lower expression levels than the nontandem genes in each of eight tissues (apical bud, young leaf, mature leaf, old leaf, young stem, root, flower, and fruit), and 26.5% of tandem genes were not expressed (FPKM < 1) in all tissues (Fig. 5c). These results revealed that the recently expanded or lineage-specific genes had lower expression, consistent with a previous report that gene expression was positively correlated with the age of gene occurrence^42^.

Furthermore, KEGG (Kyoto Encyclopedia of Genes and Genomes) enrichment analysis of tandem duplication indicated that most of these genes were related to plant–pathogen interaction (ko04626), MAPK signaling pathway (ko04016), phenylpropanoid biosynthesis (ko00940), flavonoid biosynthesis (ko00941), anthocyanin biosynthesis (ko00942), isoflavonoid biosynthesis (ko00943), etc. (Supplementary Table 7). The tandem duplication retained in a lineage-specific fashion participated in abiotic and biotic stress tolerance, suggesting that tandem duplication played an important role in adaptive evolution to rapidly changing environments and/or interaction with pathogens^40^. More than 72.5% of the nucleotide binding site (NBS) genes, an important resistance (*R*) gene family, occurred in tandem duplication. These NBS genes could be divided into six classes according to their conserved domains: N, NL, CNL, CN, TN, and RN. The majority were N type and contained only the NB-ARC domain (Fig. 5b). Expression analysis in eight tissues, a measurement of the activity and function of genes, showed that NBS genes were highly or preferentially expressed in the root (Fig. 5c), resulting from the greater pressure on the root than the other tissues^43^. Among these NBS genes, the tandem duplication did not show lower expression levels, which was inconsistent with the overall result. These highly expressed tandem duplicates of NBS genes might increase *C. sinensis* resistance to dynamic environments and pathogens. Catechin, a type of flavonoid (flavan-3-ol), is synthesized through phenylpropanoid and flavonoid biosynthesis^44,45^. KEGG analysis indicated that five categories of KEGG orthology (COMT, HCT, CCR, CAD, and E1.14.11.61) involved in phenylpropanoid biosynthesis and six (DFR, CHS, LAR, CYP75A, FLS, and C12RT1) involved in flavonoid biosynthesis were significantly enriched in tandem duplicates. Structural genes involved in the two pathways with high expression in leaves generally played important roles in governing catechin contents, such as *CsF3H*, *CsANS*, and *CsF3*ʹ*5*ʹ*H*^45,46^. Most highly expressed genes in the apical buds of these orthologs occurred in tandem duplication except for CAD, CCR, and FLS (Fig. 5d). For example, six F3ʹ5ʹH genes belonging to CYP75A that could regulate catechin contents and the ratio of di/tri-hydroxylated catechins were identified in two clusters (chr11:134.5–135.1 Mb and chr7:43.7–43.8 Mb). Of these, the highly expressed *TEA013315.1* and *TEA026296.1* have been previously cloned as *CsF3*ʹ*5*ʹ*Ha* and *CsF3*ʹ*5*ʹ*Hb*, respectively^47^ (Fig. 5e). It has been demonstrated that *TEA013315.1* plays a crucial role in the concentration of catechins and could explain the variation in catechin contents among tea germplasms^46^. Tandem duplication increased functionally divergent genes that play important roles in tea-specific biosynthesis or stress response through sub- or neofunctionalization of the retained tandem duplicates, especially through evolution of gene expression.

### Anchored QTL map

**Fig. 6:**
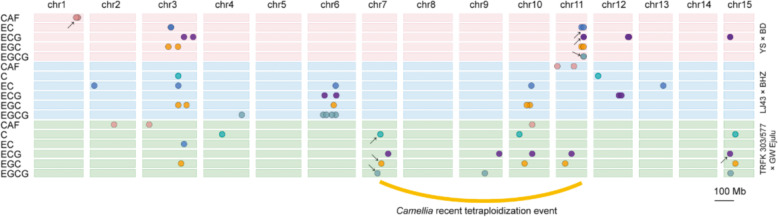
Distribution of QTLs associated with catechin and caffeine in the *C. sinensis* genome*.* CAF caffeine content, C catechin, EC epicatechin, ECG epicatechin gallate, EGC epigallocatechin, EGCG epigallocatechin gallate. The arrow represents QTLs with high phenotypic variance explained (PVE > 20%)

Catechin and caffeine, major secondary metabolites in young leaves of tea, contribute to the tea flavor and nutrient content. Among the catechins in green tea, (−)-epigallocatechin-3-gallate (EGCG) is the most abundant, followed by (−)-epigallocatechin (EGC), (−)-epicatechin-3-gallate (ECG), and (−)-epicatechin (EC)^48,49^. Based on previously reported QTLs related to the catechins and caffeine content in tea, 64 catechin- and caffeine-related QTLs were anchored to the CSS V1.2 genome (Fig. 6, Supplementary Table 8). Among these, eight caffeine-related QTLs were distributed in chr1, chr2, chr3, chr10, and chr11. The poor reproducibility of the caffeine-related QTLs identified in different experiments might be caused by the different parents used for the mapping populations. The distribution of catechin-related QTLs indicated that the EC-, ECG-, EGC-, and EGCG-related QTLs were closely linked in some regions. Six catechin-related QTL hotspots were detected on chr3, chr6, chr7, chr10, chr11, and chr15. The QTL hotspots in chr7 and chr11 showed high phenotypic variance explained (PVE > 20%). However, two QTL hotspots were not stable between the two populations of CSS ‘Yingshuang’ × *C. sinensis* var. *pubilimba* ‘Beiyue Danzhu’ and ‘TRFK 303/577’ × ‘GW Ejulu’. The analysis of WGD events implied that the regions of two hotspots were generated by the CRT event (Fig. 3, Supplementary Fig. 2). These results revealed that the QTL hotspots in chr11 might play an essential role in catechin diversification of Chinese tea germplasms, but chr7 might be responsible for tea germplasms in South Africa. In the QTL hotspot of chr11, the F3ʹ5ʹH gene *TEA013315.1* has been cloned and validated to govern catechin traits in tea plant and its relatives. The tea genome sequence will provide the basis for the polymorphic markers, genes, and repeats within these QTL regions and facilitate the identification of their effective genes.

## Discussion

The first chromosome-scale genome of a highly heterozygous tea plant was successfully accomplished by Hi-C technology. The scaffold N50 of our assembly has been increased to 218.1 Mb, which is significantly higher than that of the draft genome. Moreover, collinearity comparison with the genetic map revealed that the chromosome-scale genome presented high contiguity and accuracy of assembly. Our results demonstrated that Hi-C technology could provide a rapid, suitable, and inexpensive approach to generate a high-quality chromosome-scale assembly compared with traditional approaches, such as BAC-by-BAC and genetic maps. These traditional approaches are confronted with many challenges when used for assembly of rather complex genomes (large genome size, polyploid, and high heterozygosity) because they are laborious and expensive. In contrast, Hi-C technology has overcome the existence insufficiency and become broadly available for many complex species^50,51^. Although Hi-C technology has accelerated the completion of chromosome-scale assembly of tea plants, our assembly is not perfect due to some misjoins of small adjacent contigs and missing bases. In the future, additional high-coverage PacBio long reads will be applied to correct the misjoins and fill the gaps.

Through the analysis of WGD events in the *C. sinensis* genome, we clarified that only one recent tetraploidization event (CRT, 58.9–61.7 Mya) occurred after the ancient hexaploidization event (ECH, 146.6–152.7 Mya) shared by the eudicot genome. A phylogenetic tree of homologous genes was constructed, and it was inferred that the CRT event occurred after the divergence of *C. sinensis* and *A. chinensis* from their common ancestor (61.2–65.3 Mya)^14,36^. After polyploidization events, the genomic architecture was surprisingly varied, including massive genomic rearrangements, homologous exchange, gene loss of duplicates, proliferation of TEs, and gene expression bias of duplicates, which play an important role in the formation and evolution of species. Small RNAs, as mediators of interactions in duplicated genomes, have influences on genomic architecture that reflect their functional roles^31^. Understanding the fundamental genomic structure accelerates plant improvement and utilization. For example, in maize, ~50% phenotype- or function-associated variation is more likely to have paralogs derived from the polyploidization event than singleton genes, suggesting that gene duplication followed by neofunctionalization or subfunctionalization plays an essential role in phenotypic variation^52^. Our observation that extraordinary variation in genome size, gene loss, gene expression, and tandem duplicates burst after the CRT event. Two QTL hotspots in chr11 and chr7 derived from the CRT event might be responsible for catechin diversification of the tea germplasms.

Tea is one of the most popular nonalcoholic beverages in the world and has numerous health benefits for humans. The availability of a chromosome-scale genome holds great promise for the understanding of fundamental genomic architecture and evolution, and for the improvement of tea plant quality (such as catechin, theanine and caffeine) and yield. Integrated with GWAS, map-based clone, and bulked segregant analysis, the chromosome-scale genome will accelerate the identification of trait-related variations or genes that can be used for the rapid, effective, and inexpensive selection of available germplasms or cultivars.

## Materials and methods

### Hi-C sequencing and genome sequence

Fresh leaves of CSS ‘Shuchazao’ grown at the China National Germplasm Tea Repository (Hangzhou, Zhejiang, China) were selected for Hi-C sequencing. An in situ Hi-C library based on DpnII was constructed as described previously^53^ and the resulting library with an insert size of ~300 bp was sequenced on a HiSeq 4000 under 2 × 150 bp mode. The draft genome sequence of CSS ‘Shuchazao’ was downloaded from the Tea Plant Information Archive (TPIA, http://tpia.teaplant.org/)^13^. The gene annotation and TE annotation used in this study were also obtained from the draft genome sequence of CSS ‘Shuchazao’^13^.

### Chromosome assembly with Hi-C

Raw Hi-C reads were mapped to the draft genome sequence using bwa (version 0.7.17-r1188)^54^, and then erroneous mappings (MAPQ = 0) and duplicates were filtered by the Juicer pipeline^55^. The output of the Juicer pipeline was used for 3D-DNA^56^ analysis with default parameters, including misjoin correction, ordering, and orientation. To ensure the accuracy of assembly, the scaffold misjoins, ordering, and orientation were further checked and corrected manually based on the interaction matrix of scaffolds from the Juicebox visualization system^57^.

### Comparison of genetic map and physical map

To assess the CSS V1.2 genome, two public genetic maps^22,41^ were used for collinearity analysis compared with the chromosome-scale assembly. A total of 6042 valid SNP markers of a SLAF-seq genetic map (F1 population, CSS ‘Yingshuang’ × *C. sinensis* var. *pubilimba* ‘Beiyue Danzhu’)^22^ and 2380 available markers of a 2b-RAD genetic map (F1 population, CSS ‘Longjing 43’ × CSS ‘Baihaozao’)^41^ were aligned to the assembled genome by Bowtie (version 1.2.1.1)^58^ with ‘-a –v 1’ parameters. To obtain unambiguous alignments, reads that mapped to more than two places in the genome were removed. The dotplot of collinearity comparison was drawn by Perl script with the SVG module.

### Analysis of WGD and tandem duplication

Genes anchored to chromosomes in the *C. sinensis*, *V. vinifera*, and *A. chinensis* genomes were used to analyze genome evolution. First, protein sequences of *C. sinensis* were searched against the three genomes to find potentially homologous genes using BLASTP^59^ (*e*-value threshold, 1e−5). Second, large gene families with >30 matches were filtered out, and the remaining homologous genes were used for inferring homologous blocks by MCScanX^60^ software with ‘-s 4 –m 50 –w 5’ parameters. The synonymous nucleotide substitution rates (*K*s) were calculated by using *add_ka_and_ks_to_collinearity.pl* implemented in MCScanX.

To distinguish the event-related homologous blocks, homologous blocks between the *C. sinensis* and *V. vinifera* genomes attributable to WGD events occurring after the tea-grape split were identified by the criterion that the best-matched homologous gene pairs accounted for >50% in a homologous block. To identify the ECH and CRT events in the *C. sinensis* genome, any homologous block with median *K*s > 0.7 was defined as an ECH event, and any other was defined as a CRT event. The smooth curve of *K*s distribution was obtained by using the Gaussian kernel function.

To identify tandem duplications, all genes anchored to the chromosomes were compared by BLASTP to find homologous genes (*e*-value < 1e−20). The homologous genes with a maximum of five intervening genes were defined as tandem duplicates^61^. KEGG enrichment analysis of tandem duplicates was performed by clusterProfiler package^62^ with the cutoff set at adjusted *P* value < 0.05.

### RNA-seq data analysis

The RNA-seq data of eight tissues (apical bud, young leaf, mature leaf, old leaf, young stem, root, flower, and fruit) of CSS ‘Shuchazao’ were obtained from the NCBI SRA database submitted by Wei et al.^13^ (Supplementary Table 9). The raw Illumina reads were trimmed by Trimmomatic (v 0.36)^63^ and then aligned to the CSS V1.2 reference genome using HISAT2^64^ with default settings. Differential expression between duplicate genes was calculated by the edgeR package^65^ with an FDR < 0.05 and at least a two-fold difference in expression levels.

### Identification of NBS genes

All genes from the *C. sinensis* genome were annotated using HMMER 3.1b2 (http://hmmer.org/) against the Pfam database with an *e*-value threshold of 0.001. Genes with NB-ARC domains (PF00931) were defined as NBS genes and used for further analysis. These NBS genes were classified by different conserved domains identified by Pfam and the NCBI Conserved Domains Tool^66^. The conserved domains were confirmed using the following accession numbers: TIR (PF13676, PF01582), LRR (PF00560, PF07723, PF07725, PF12799, PF13306, PF13516, PF13504, PF13855, cl34836), and RPW8 (PF05659). CC domains were detected and confirmed by Paircoil2^67^ with a *P*-score cutoff of 0.025.

## Supplementary information

Supplementary Figure S1-4 and Table S1, S2, S4, S5, S6, S9

Supplementary Table S3, S7, S8

## Data Availability

The raw Hi-C data of CSS ‘Shuchazao’ have been deposited in the NCBI Sequence Read Archive (SRA) under BioProject accession no. PRJNA596054. The CSS V1.2 assembly data is available at https://github.com/JiedanChen/TeaGenomeData.
