# Supplementary material for: The chromosome-scale genome reveals the evolution and diversification after the recent tetraploidization event in tea plant
Source: Hortic Res. 2020 May 1;7:63. doi: 10.1038/s41438-020-0288-2 (PMC7192901; doi:10.1038/s41438-020-0288-2)
Supplement: Supplementary file 1 — Supplementary Figure S1-4 and Table S1, S2, S4, S5, S6, S9 [file 41438_2020_288_MOESM1_ESM.docx]

Supplementary Information


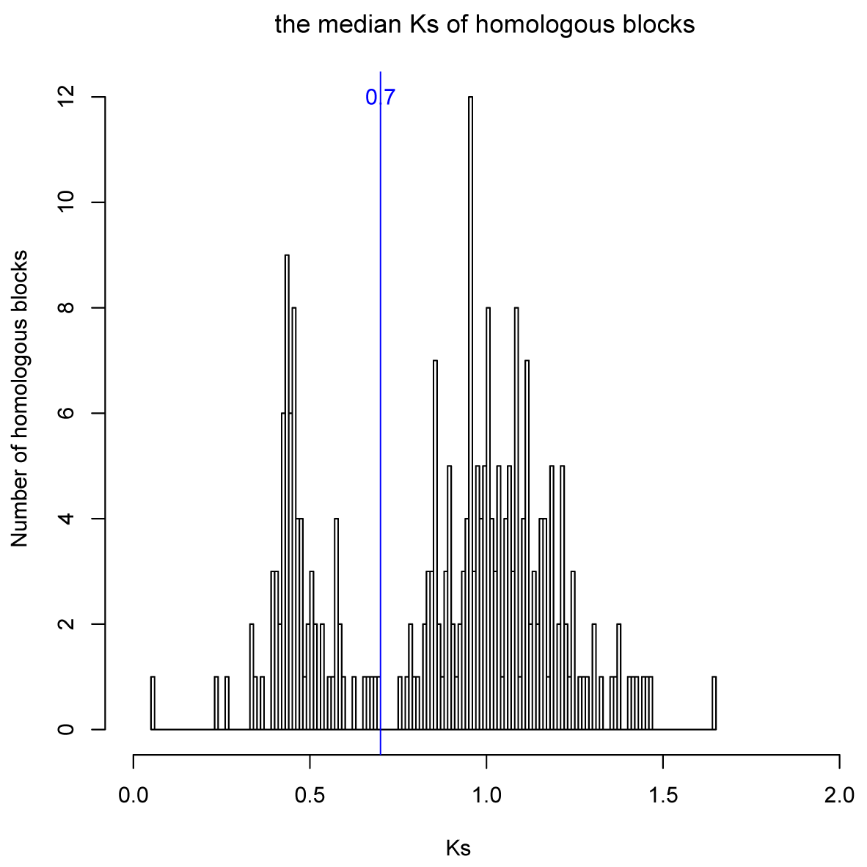


**Figure S1.** Distribution of the median Ks of 254 homologous blocks within *C. sinensis* genome.

The value 0.7 of median Ks of homologous block was chose to identify CRT-related homologous blocks and ECH-related homologous blocks. CRT: *Camellia* recent tetraploidization, ECH: core-eudicot common hexaploidization.


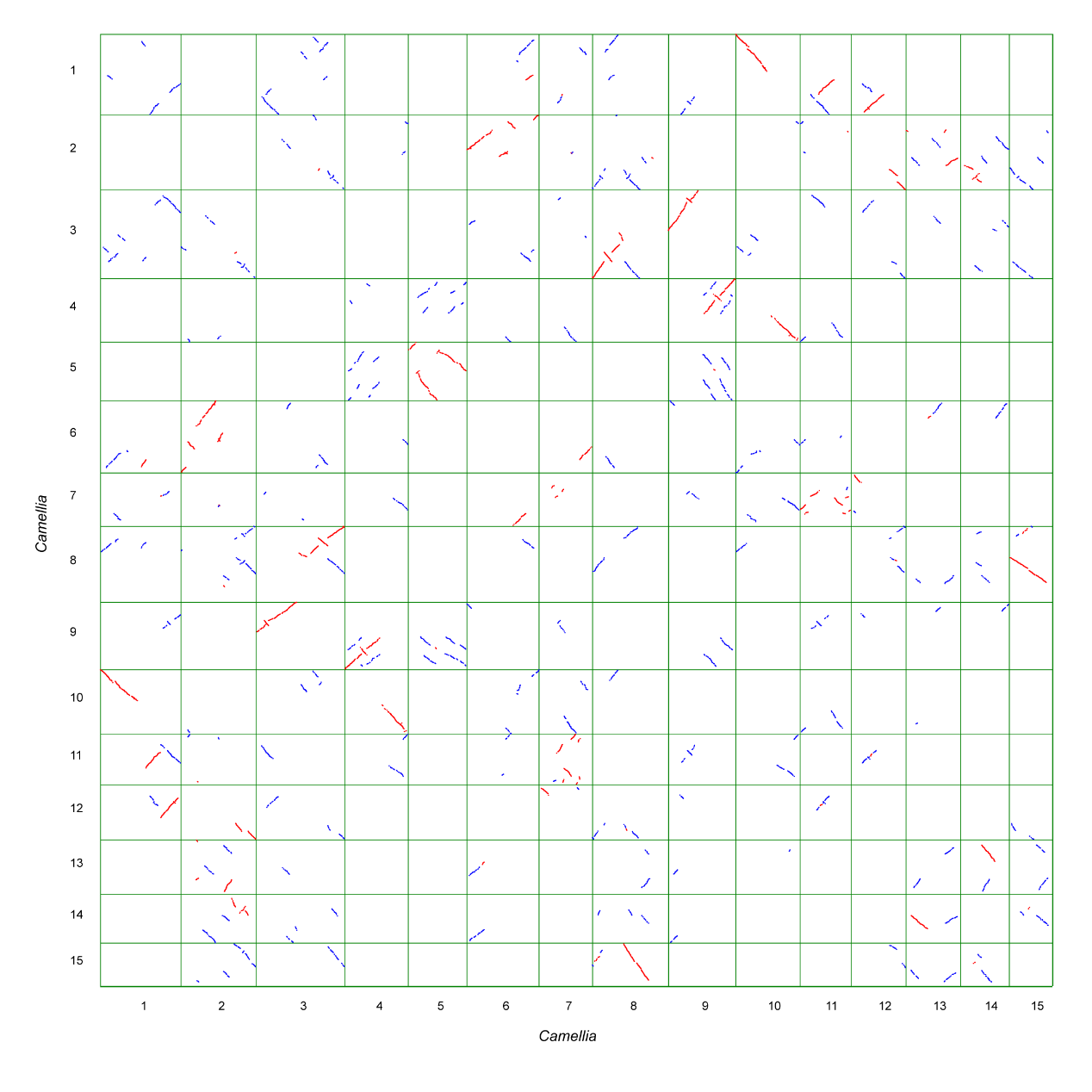


**Figure S2.** Homologous gene dotplot within *C. sinensis* genome.

The median Ks of homologous blocks <= 0.7 are in red and >0.7 in blue, representing the *Camellia* recent tetraploidization (CRT) event and core-eudicot common hexaploidization (ECH) event, respectively.


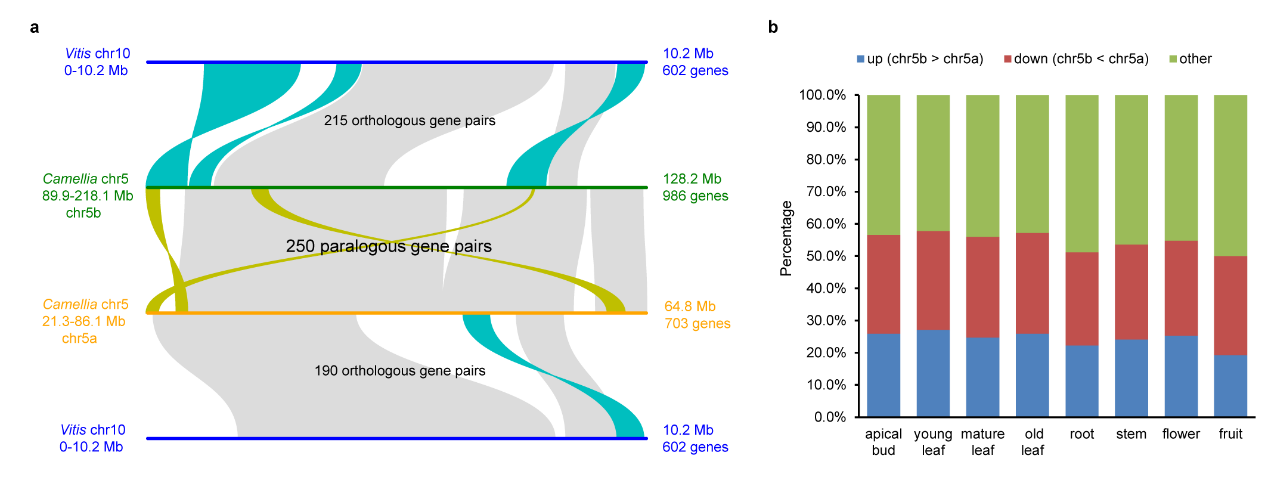


**Figure S3.** The diversification of homologous genes generated by the CRT event in *C. sinensis* chr5.

(a) Analysis of the synteny within *C. sinensis* chr5 and among genomes. Blue links indicate inversion and translocation between the *C. sinensis* and *V. vinifera* genomes. Yellow links indicate inversion and translocation within *C. sinensis* chr5 that occurred after the CRT event; (b) The expression divergence between duplicate genes generated by the CRT event in *C. sinensis* chr5.


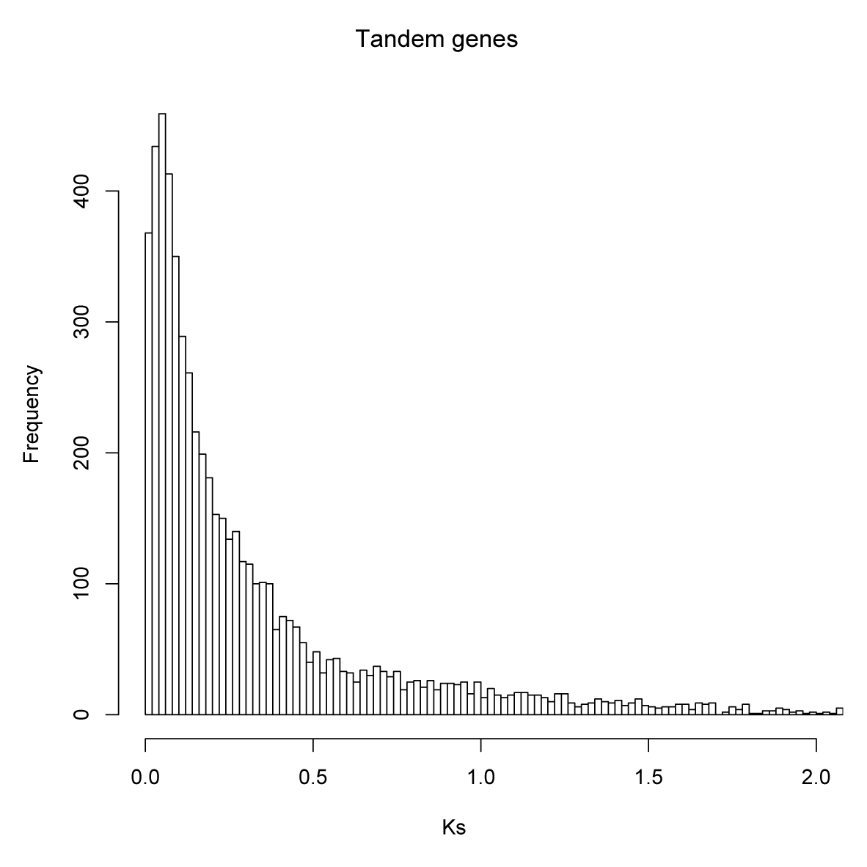


**Figure S4.** Distribution of Ks divergence of tandem gene pairs in clusters with exactly two tandem genes.

**Table S1.** Comparison of the *C. sinensis* var. *sinensis* original and improved (CSS V1.2) assembly.

|  | **Original assembly** | **CSS V1.2 genome** |
| --- | --- | --- |
| **Scaffold length (bp)** | 3,141,536,798 | 3,152,597,298 |
| **Scaffold number** | 14,051 | 14,412 |
| **Scaffold N25 (bp)** | 2,330,095 | 228,659,840 |
| **Scaffold N50 (bp)** | 1,397,810 | 218,115,851 |
| **Scaffold N75 (bp)** | 762,138 | 178,023,153 |
| **Scaffolds anchored to chromosomes (bp)** | - | 2,984,696,770 (94.7%) |

**Table S2.** Summary of the repetitive sequences and genes of each chromosome in the CSS V1.2 genome.

|  | **Length (bp)** | **Repetitive Sequences (bp)** | **Repetitive Sequences (%)** | **No. Gene** | **Average Length of Genes** |
| --- | --- | --- | --- | --- | --- |
| **chr1** | 237,570,591 | 137,081,442 | 57.7% | 2,729 | 1333.8 |
| **chr2** | 218,144,730 | 123,066,685 | 56.4% | 2,549 | 1327.5 |
| **chr3** | 264,061,170 | 153,991,820 | 58.3% | 3,013 | 1369.7 |
| **chr4** | 220,359,316 | 126,556,471 | 57.4% | 2,154 | 1332.4 |
| **chr5** | 218,115,851 | 129,717,791 | 59.5% | 1,990 | 1323.8 |
| **chr6** | 228,659,840 | 127,604,120 | 55.8% | 2,449 | 1334.3 |
| **chr7** | 160,918,114 | 90,760,657 | 56.4% | 1,816 | 1313.8 |
| **chr8** | 237,966,957 | 136,940,488 | 57.5% | 2,578 | 1311.7 |
| **chr9** | 205,531,979 | 119,895,956 | 58.3% | 2,286 | 1333.5 |
| **chr10** | 191,021,555 | 107,394,464 | 56.2% | 2,180 | 1358.4 |
| **chr11** | 143,513,403 | 83,405,702 | 58.1% | 1,735 | 1376.8 |
| **chr12** | 184,378,419 | 105,922,837 | 57.4% | 1,860 | 1332.9 |
| **chr13** | 156,635,078 | 86,995,268 | 55.5% | 1,854 | 1331.8 |
| **chr14** | 178,023,153 | 104,809,330 | 58.9% | 1,651 | 1355.7 |
| **chr15** | 139,796,614 | 81,073,509 | 58.0% | 1,467 | 1377.8 |
| **UN** | 167,900,528 | 94,047,363 | 56.0% | 459 | 1039.2 |
| **Total** | 3,152,597,298 | 1,809,263,903 | 57.4% | 32,770 | 1335.8 |

**Table S4.** The statistic of event-related homologous blocks within *C. sinensis*, *V. vinifera* and *A. chinensis* genomes.

| **Within a genome** | **Homologous blocks** | **Gene pairs** | **Average collinear gene pairs per block** |
| --- | --- | --- | --- |
| ***Camellia* CRT-related** | 77 | 2,596 | 33.71 |
| ***Camellia* ECH-related** | 177 | 2,152 | 12.16 |
| ***Actinidia* ART-related** | 239 | 4,028 | 16.85 |
| ***Actinidia* AAT-related** | 217 | 2,251 | 10.37 |
| ***Actinidia* ECH-related** | 177 | 1,356 | 7.66 |
| ***Vitis* ECH-related** | 107 | 1,582 | 14.79 |

**Table S5.** The statistic of homologous blocks within a genome or between genomes.

| **Within a genome or between genomes** | **Anchored genes** | **Homologous blocks** | **Gene pairs** | **Average colinear gene pairs per block** | **Homologous genes** |
| --- | --- | --- | --- | --- | --- |
| ***Camellia*** | 32,311 | 254 | 4,748 | 18.69 | 6,677 |
| ***Vitis*** | 23,647 | 107 | 1,582 | 14.79 | 2,720 |
| ***Actinidia*** | 30,906 | 633 | 7,635 | 12.06 | 10,138 |
| ***Actinidia* vs *Vitis*** | 23,647 vs 30,906 | 1,058 | 14,259 | 13.48 | 10,987 vs 10,671 |
| ***Camellia* vs *Vitis*** | 32,311 vs 23,647 | 714 | 14,831 | 20.77 | 11,411 vs 9,213 |
| ***Camellia* vs *Actinidia*** | 32,311 vs 30,906 | 1,394 | 22,106 | 15.86 | 11,325 vs 13,969 |

**Table S6.** Tandem gene statistics of *C. sinensis*, *V. vinifera* and *A. chinensis* genomes.

| **Species** | **Number of tandem expanded regions** | **Total number of retained tandem genes (%)** |
| --- | --- | --- |
| ***C. sinensis*** | 3,262 | 9,243 (28.6%) |
| ***V. vinifera*** | 1,729 | 5,088 (21.5%) |
| ***A. chinensis*** | 1,343 | 3,111 (10.1%) |

**Table S9.** The RNA-seq data of *C. sinensis var. sinensis* used in this study.

| **Tissue** | **SRA ID** | **Bases (Gb)** | **Cultivar** | **BioProject** |
| --- | --- | --- | --- | --- |
| **young leaf** | SRR7474026 | 11.22 | Shuchazao | PRJNA274203 |
| **apical bud** | SRR7474027 | 12.21 | Shuchazao | PRJNA274203 |
| **old leaf** | SRR7474028 | 12.02 | Shuchazao | PRJNA274203 |
| **mature leaf** | SRR7474029 | 10.79 | Shuchazao | PRJNA274203 |
| **flower** | SRR7474030 | 12.43 | Shuchazao | PRJNA274203 |
| **young stem** | SRR7474031 | 11.58 | Shuchazao | PRJNA274203 |
| **root** | SRR7474032 | 12.35 | Shuchazao | PRJNA274203 |
| **fruit** | SRR7474033 | 11.52 | Shuchazao | PRJNA274203 |
